# Effect of the gut microbiota and their metabolites on postoperative intestinal motility and its underlying mechanisms

**DOI:** 10.1186/s12967-023-04215-2

**Published:** 2023-05-26

**Authors:** TianRong Ma, XiaoLei Xue, Hui Tian, XinXiu Zhou, JunKe Wang, ZhiWen Zhao, MingFei Wang, JiYuan Song, RenXiang Feng, Leping Li, Changqing Jing, Feng Tian

**Affiliations:** 1 Department of Gastrointestinal Surgery, Shandong Provincial Hospital Affiliated to Shandong First Medical University, Jinan, 250021 China; 2 Department of Gastrointestinal Surgery, Shandong Provincial Hospital, Cheeloo College of Medicine, Shandong University, Jinan, 250021 China; 3 Department of Pharmacy, The Second Affiliated Hospital of Shandong First Medical University, Taian, 271000 China; 4 Department of Gastroenterology, Liaocheng People’s Hospital, Shandong First Medical University, Liaocheng, 252000 China

**Keywords:** Gut microbiota, Intestinal motility, Metabolites, Postoperative ileus, Probiotics, Review

## Abstract

Gut microbiota is closely related to human health and disease because, together with their metabolites, gut microbiota maintain normal intestinal peristalsis. The use of antibiotics or opioid anesthetics, or both, during surgical procedures can lead to dysbiosis and affect intestinal motility; however, the underlying mechanisms are not fully known. This review aims to discuss the effect of gut microbiota and their metabolites on postoperative intestinal motility, focusing on regulating the enteric nervous system, 5-hydroxytryptamine neurotransmitter, and aryl hydrocarbon receptor.

## Introduction

Postoperative ileus (POI) refers to the prolonged recovery time of the gastrointestinal tract after surgery, resulting in abdominal distension, vomiting, oral intolerance, and delayed bowel function [1]. At least one in eight patients develops POI following elective intestinal surgery [2]. The pathogenesis of POI is manifested in different stages, namely, a short-acting neurogenic phase marked by exaggerated inhibitory reflexes and a longer inflammatory phase [3]. The duration of POI is influenced by the degree of surgical trauma, which is most extensive following intestinal surgery, however, POI can occur after any surgery [4]. Several strategies have been proposed to reduce POI, such as multimodal postoperative rehabilitation (fast-track care) and minimally invasive surgery, although their ability to shorten POI duration is limited [5]. Therefore, POI is an important factor affecting the postoperative recovery of patients. Early recovery of the postoperative intestinal function cannot only improve the patient’s clinical outcome and quality of life, especially for patients that have undergone gastrointestinal surgery, but also shorten the hospital stay, increase the turnover of hospital beds, and save medical resources. POI is related to anesthetic use, surgical stress, inflammatory responses, intestinal autonomic nervous system injury, and gastrointestinal disorders [6].

The gut of healthy adults contains approximately 10^12^–10^14^ microorganisms [7], present throughout the gastrointestinal tract but most dense and diverse in the colon. Gut microbiota can maintain the dynamic balance in the host and regulate inflammation and the immune system via host interactions [8]. The relationship between gut microbiota and postoperative intestinal recovery has attracted much attention. Assessing the relationship between gut microbiota and postoperative intestinal motility can provide a theoretical basis for the recovery of postoperative intestinal function. Gut microbiota may play an important role in the neurogenic phase and inflammatory counterpart of POI. An imbalance in the host gut microbiota has been established as a cause of intestinal dysfunction. In the present review, the critical role of the gut microbiota in intestinal motility recovery following gastrointestinal surgery is presented, and the latest research on elucidating the mechanisms is discussed. The information presented here can provide treatment guidelines for postoperative intestinal motility issues.

## Changes in the gut microbiota and intestinal motility after surgery

The gut microbiota play a crucial role in the intestinal function. Under normal conditions, beneficial microbiota with normal abundance in the intestine interacts with pathogens present at low abundance to prevent them from crossing the intestinal barrier [9]. However, the gut microbiota change upon being subjected to surgical stresses, such as anesthesia, perioperative antibiotics, and surgery. In particular, after severe physiological damage, the density and function of gut microbiota are altered [10]. For example, colectomy analysis detected bacterial translocation, and the abundances of *Enterococcus* and *Escherichia*, and *Shigella* increased 500- and 200-fold, respectively [11].

**Fig. 1:**
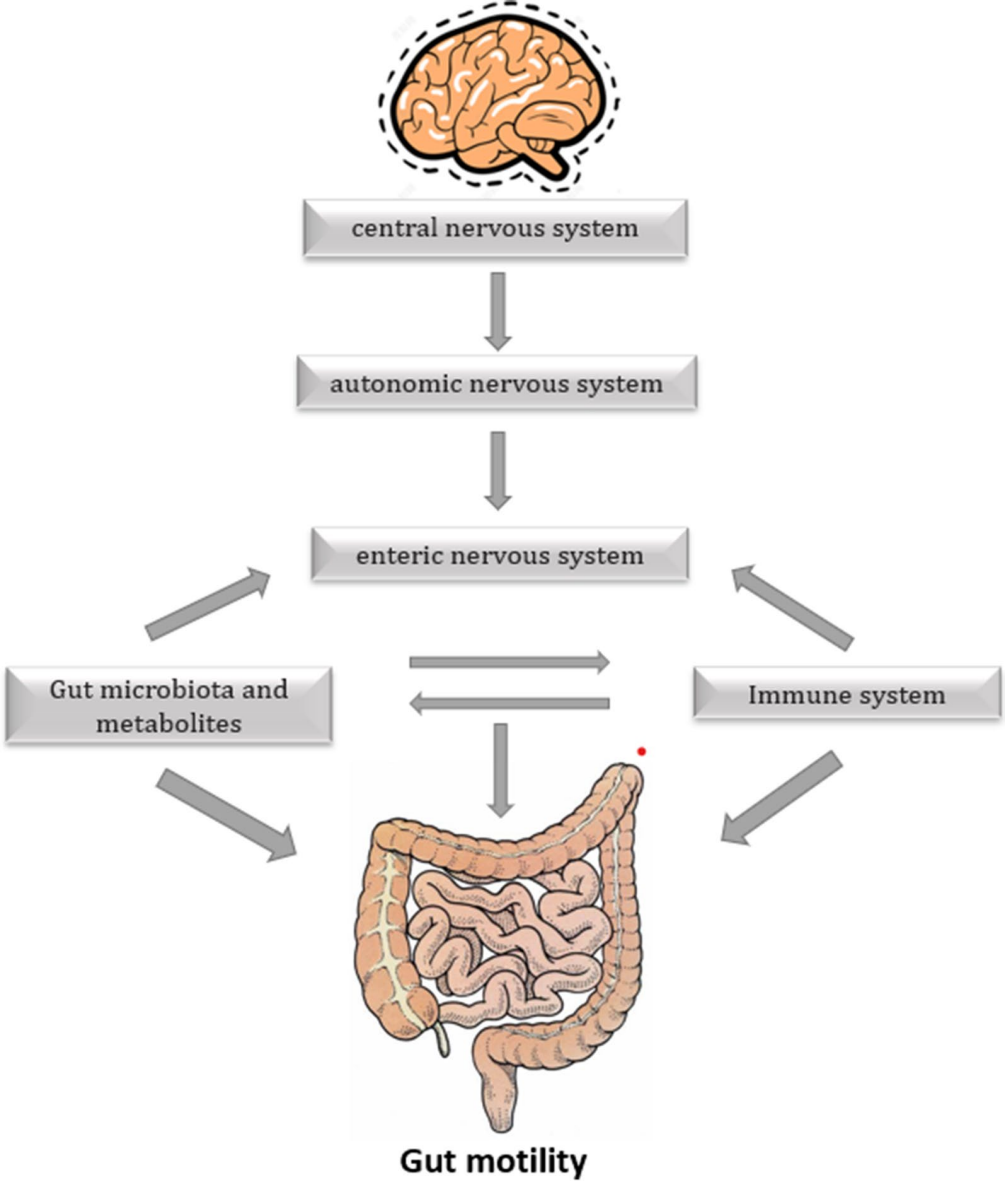
Postoperative gut microbiota and changes in microbiota metabolites that affect intestinal motility. Surgery may lead to gut microbiota disorders and changes in the levels of their metabolites, such as bile acid (BA), short-chain fatty acids (SCFAs), and indole propionic acid (IPA). The underlying mechanisms of these changes include the following. Dendritic cells (DC) are induced to produce interleukin (IL)-12 and T helper type I (Th1) cells, which secrete interferons (IFNs), thereby activating mast cells (MCs) and muscle macrophages (MMs) to generate pro-inflammatory cytokines, such as nitric oxide (NO), tumor necrosis factor (TNF), and IL-1β. It inhibits tryptophan hydroxylase 1 (TPH1) production of 5-hydroxytryptamine (5-HT) in enterochromaffin cells (ECs), and 5-HT can regulate the release of neurotransmitters, such as substance P. Activation of receptors on the surface of neurons, such as Toll-like receptor (TLR)2/TLR4, can be directly suppressed. Activation of neuronal nuclear receptors, such as aryl hydrocarbon receptor (AHR), can be suppressed to inhibit the transcription of neurotransmitters

Moreover, gastrointestinal surgery increases intestinal permeability and destroys the intestinal barrier function, leading to an imbalance in intestinal microbiota and bacterial translocation [12]. *Bacteroides* in the gut can stimulate intestinal peristalsis by increasing the expression of γ-aminobutyric acid, vesicle-associated protein, and intestinal γ-actin [13, 14]. γ-aminobutyric acid, as a neurotransmitter, can act on neurons and participates in colon peristalsis [15]. Other beneficial bacteria, such as *Lactobacillus acidophilus* and *Bifidobacterium*, can promote intestinal transport by releasing neurotransmitters. In contrast, pathogenic bacteria, such as *Micrococcus flavus* and *Escherichia coli*, can impair the contraction of colonic muscle cells and inhibit intestinal transport [16]. Studies have shown that after sleeve surgery, the level of the Gammaproteobacteria class was higher, while the level of the Firmicutes phylum was lower [17, 18]. Firmicutes are considered to inhibit intestinal inflammation [19]. In an intestinal obstruction model, the abundance of Firmicutes decreased [20]. Although patients who have undergone surgery, especially gastrointestinal surgery, exhibit substantial shifts in bacterial composition, the structural disturbance of gut microbiota detected in the early postoperative period returned to the baseline after an average of 31 days [21]. Changes in gut microbiota caused by preoperative and intraoperative intestinal preparation could lead to a decline in intestinal motility. During the development of intestinal obstruction, gut microbiota might affect intestinal motility by changing the levels of derived metabolites, such as bile acids (BAs) and short-chain fatty acids (SCFAs) [22–24] (Fig. 1).

Laxatives and prophylactic antibiotics are often used in gastrointestinal surgery, frequently leading to disorders of gut microbiota and intestinal motility dysfunction. After antibiotic treatment, the abundance of the intestinal microbiota significantly decreases [25]. In germ-free animals, antibiotic-induced imbalance in the gut microbiota led to a delay in gastrointestinal transport, gastric emptying, and intestinal tract motivation damage [26]. The preoperative use of opioid anesthetics can also affect intestinal motility and gut microbiota. Both exogenous and endogenous opioids have immunosuppressive effects. They can change the microbial function [27], causing bacterial translocation and increasing the expression of pro-inflammatory cytokines, such as interleukin (IL)-1β, in colonic tissues. In addition, opioids can disturb BA metabolism, destroy the intestinal barrier, and affect intestinal motility [28]. Moreover, activating opioid receptors in the intestine can directly affect intestinal motility, as has been demonstrated for loperamide [29, 30].

**Fig. 2:**
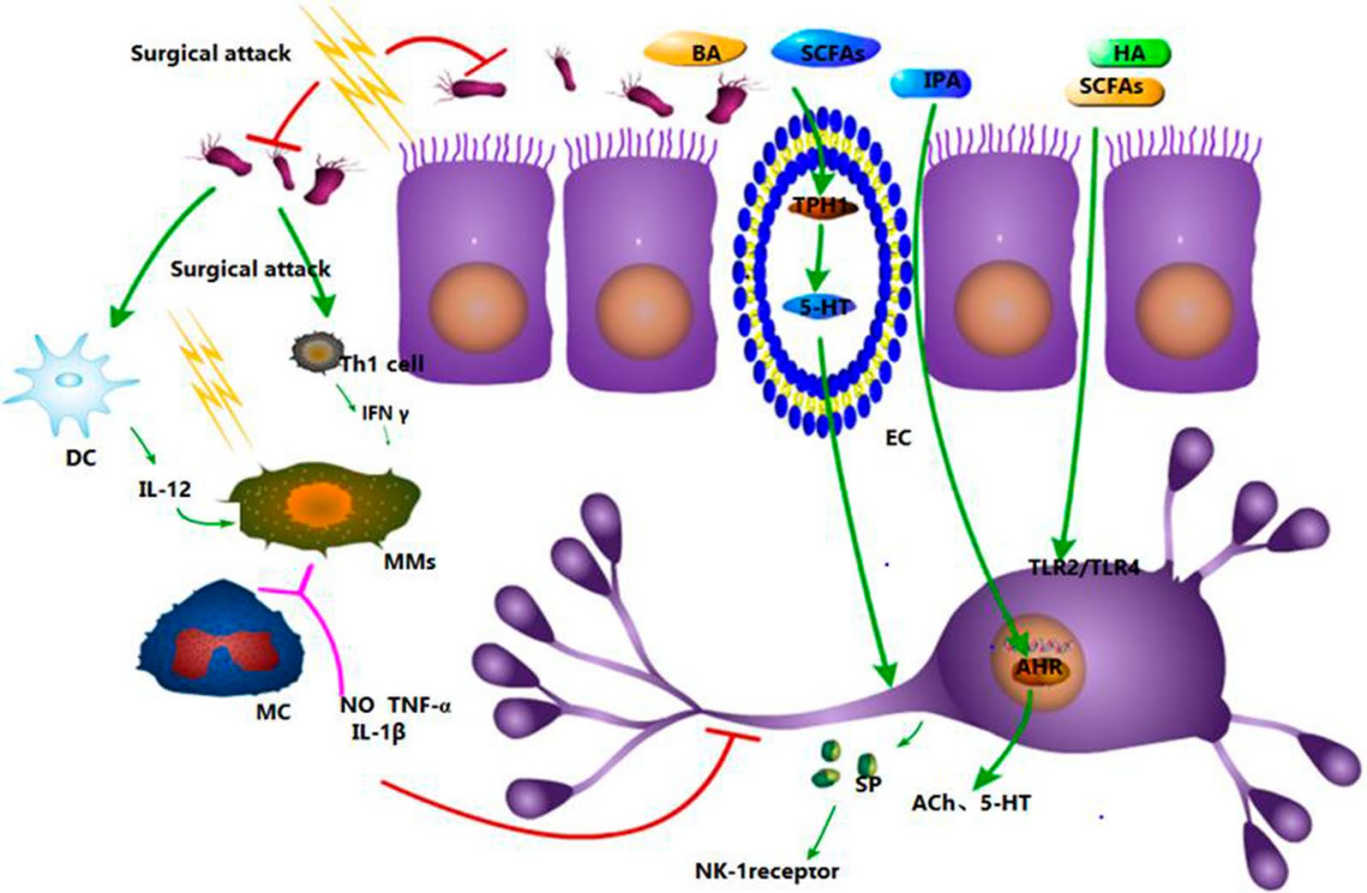
Schematic diagram of postoperative gut microbiota and metabolites changes impaired intestinal motility. The surgical attack will cause gut microbiota disorder and changes in metabolites such as BA, SCFAS and IPA. The changes in gut microbiota and its metabolites can be achieved by (1) inducing DC cells to produce IL-12 and TH1 cells to produce IFN, activating MC and MMs to produce proinflammatory cytokines such as NO, tnf and IL-1 β; (2) it inhibits TPH1 production of 5 HT in EC cells, and 5 HT can regulate the release of neurotransmitters such as sp; (3) directly reduce the activation of receptors on the surface of neurons, such as TLR2/TLR4; (4) directly reduce the activation of neuronal nuclear receptors such as AHR and inhibit the transcription of neurotransmitters

Opioid anesthetics can inhibit peristalsis and induce an imbalance in gut microbiota; however, no clear evidence exists suggesting that microbiota imbalance is involved in intestinal motility inhibition after anesthesia; thus, relevant results must be further studied (Fig. 2).

## Effects of gut microbiota and their metabolites on postoperative intestinal motility via the enteric nervous system

The microbiota gut brain axis is a two-way system. The enteric nervous system (ENS) maintains intestinal health and peristalsis by regulating gut microbiota, which is essential in the development of the gastrointestinal and nervous systems, including the ENS. Moreover, the fermentation products of gut microbiota promote the synthesis of various neurotransmitters and regulate the secretion of signaling molecules [31, 32], which play key roles in developing and promoting intestinal neuromotor function. Under steady-state conditions, the gastrointestinal function depends on the interaction between the ENS and gut microbiota. However, the physiological pressure of surgery, antibiotic treatment, and surgical injury can alter the gastrointestinal environment, leading to an imbalance in gut microbiota. In addition, damage to the anatomical structure and functional integrity of the intestine may result in systemic inflammation and bacterial translocation, further influencing intestinal motility.

### Gut microbiota regulate intestinal motility via enteric neurons and glial cells

The ENS is mainly distributed in the myenteric and submucosal plexuses; however, intestinal motility is solely controlled by the myenteric plexus [33]. The ENS comprises neurons and enteric glial cells [34]. Intestinal smooth muscle cells are controlled by excitatory and inhibitory motor neurons [35] that can generate electrical activity, resulting in slow wave peristaltic movements [36]. Peristalsis is heavily dependent on the ENS and cannot occur when ENS activity is absent [37].

To perform its normal functions, each nerve plexus must contain an appropriate density of ganglia and a sufficient proportion of neuronal subtypes and glial cells in each ganglion [38]. Enterectomy can reduce microbial diversity in the residual intestine and colon, leading to neuronal damage. The decrease in the number of ganglion cells beyond a certain level results in an imbalance in the proportion of intermuscular nerves and increases the number of nitrogenous neurons, leading to decreased intestinal motility [39]. However, the underlying mechanism remains unclear. Mice administered with IL-1 receptor antagonist anakinra or antibodies to deplete IL- 1α and IL- 1β before intestinal manipulation were protected from POI [40]. The release of Ca^2+^ from the endoplasmic reticulum is a critical regulatory step in coupling IL-1 signaling [41]. The change in Ca^2+^ concentration due to IL-1 signaling may regulate intestinal motility and transport. However, surgical elimination of enteric glial cells is likely to damage the ENS and intestinal motility and can cause epithelial cell imbalance, leading to bacterial translocation and intestinal inflammation.

### Gut microbiota affect intestinal motility by regulating Toll-like receptors (TLRs)

TLRs, an important family of pattern recognition receptors involved in non-specific immunity (natural immunity), can recognize pathogens triggering immune responses that affect intestinal function. The mechanisms by which gut microbiota affect intestinal motility by regulating TLRs are complex. Gastrointestinal surgery can disrupt gut microbiota. Due to the intestinal barrier dysfunction caused by surgery, gut microbiota can be transferred to the muscularis externa (ME) [42, 43]. The translocated gut microbiota exhibit multiple TLR ligands that bind with several TLRs expressed by resident ME macrophages [44]. Evidence from a POI mouse model with TRIF/TLR3 deletion suggests that this deletion reduces ME inflammation and protects against POI [45]. Following gastrointestinal surgery, the TLR3 ligand expressed by the translocated gut microbiota can activate the TLR3/TRIF axis, promoting the production of proinflammatory cytokines in macrophages, ultimately leading to POI. Moreover, the disturbance of gut microbiota caused by gastrointestinal surgery may affect the production of ligands for TLR, such as TLR2 and TLR4. TLR2 and TLR4 are expressed in the enteric neurons, glial cells, and smooth muscle cells and can regulate intestinal motility [46, 47]. In mice treated with antibiotics, intestinal motility was inhibited, bowel movement frequency was reduced, and the total number of neurons decreased. These observations are similar to those in mice lacking TLR2 [47]. Neurochemical coding of enteric neurons and smooth muscle glial cell line-derived neurotrophic factor were abnormal in germ-free mice deficient in TLR2 signaling [48]. Moreover, mice lacking gut microbiota showed an ENS deficiency that could be prevented by administering TLR2 agonists, suggesting that TLR2 signaling regulates intestinal inflammation by controlling the structure and neurochemical coding of the ENS and intestinal neuromuscular function [48]. Activation of TLR2 in the smooth muscles led to the production of neurotrophins, thereby enhancing the structural and functional integrity of the intestinal nervous system [49]. TLR4 both improves and delays intestinal movement. Under normal physiological conditions, interactions between bacterial particles, such as lipopolysaccharides (LPS), and enteric neurons have been shown to improve the survival rate of enteric neurons and intestinal motility in *TLR4*-knockout mice. Concurrently, LPS-induced TLR4 activation promotes neuronal survival and improves intestinal motility. In contrast, the lack of TLR4 signaling delays intestinal peristalsis [50]. However, TLR4 could inhibit intestinal movement following surgery. Studies have also shown that postoperative TLR4- mice had stronger contractile activity of intestinal smooth muscles post-operation than wild mice, and their jejunum muscle was not prone to dysfunction following surgery [51, 52]. Moreover, the levels of TNF-α in the intestine and plasma were significantly decreased in TLR4^−^ mice. These results suggest that TLR4 promotes inflammatory response by activating the mitogen-activated protein kinase signaling pathway, thereby leading to POI.

### Gut microbiota influence the ENS by regulating inflammation

Inflammation can affect the ENS, resulting in intestinal dyskinesia. Antibiotic-induced microbiota community depletion in mice can result in low-grade intestinal inflammation, reduced intestinal transport time, and spontaneous contraction amplitude [53]. Intestinal abdominal surgery can cause inflammatory reactions in intestinal cells, resulting in the expression of inducible nitric oxide synthase (iNOS), IL-6, and IL-1β; rapid increase in pro-inflammatory mediators and long-term dysfunction of intestinal motility may lead to postoperative intestinal obstruction [54].

Mast cells (MCs) regulate intestinal motility, and acute or chronic stress can induce MC activation [55]. Infiltration and activation of MCs interfere with intestinal motility and play an important role in regulating the kinetic energy of intestinal myometrial neurons and smooth muscle [56, 57]. MCs also play an important role in POI; MC infiltration is significantly increased in the early stage of POI [58]. During intestinal surgery, MCs degranulate and induce the release of mediators, which leads to the formation of local infiltration in the intestinal wall. This inflammatory process influences the occurrence and maintenance of POI by affecting the gastrointestinal movement of the non-operative intestinal segment [59]. In addition, maintaining MC stability and macrophage inhibition can improve the inflammatory dynamic disorder of POI [58].

The microenvironment of the intestinal lumen, including symbiotic bacteria and their products, plays a crucial role in regulating the activation and secretion of MCs [57]. When the intestinal microbiota is imbalanced, some fungi can activate MCs via TLRs and other physical and chemical factors to release histamine, chemokines, and lipid metabolites. These compounds participate in intestinal inflammation, lead to intestinal dysfunction, and affect intestinal peristalsis [60]. Evidence shows that gut microbiota is maladjusted, and the microbial diversity is reduced in patients with irritable bowel syndrome, which could lead to MC degranulation and affect intestinal motility [61, 62].

Muscle macrophages (MMs) are highly heterogeneous phagocytes that actively contribute to organ homeostasis and are abundant in different gastrointestinal tract tissues [63]. In a POI mouse model, chemical or genetic depletion of MMs significantly improved the inhibition of smooth muscle and intestinal motility disorders [64], while a study on short bowel syndrome revealed that, in rats undergoing small bowel resection, the total number of macrophages, levels of the inflammatory marker iNOS, and proportion of nitrated neurons in the jejunum increased, while intestinal motility decreased [65]. These findings suggest that MMs participate in local inflammatory reactions, leading to POI.

In the outer muscular layer, MMs are located in a dense network in the myenteric plexus of the ENS [66]. Neuron-associated MMs in the myenteric plexus are critical to the survival of myenteric neurons; their depletion leads to caspase-3-mediated apoptosis and loss of more than 50% of the neurons in the myenteric nerves, resulting in impaired peristalsis and prolonged intestinal transmission [67]. Bone morphogenetic protein 2 (BMP2) could be generated by neuron-associated MMs, critical for regulating enteric neurons and peristalsis [66]. Surgery leads to the activation of MMs, triggering an inflammatory cascade in the outer muscle layer and inducing the expression of pro-inflammatory genes and recruitment of chemokines and cytokines, which was related to the temporary impairment of intestinal motility in both mice and humans [68, 69]. In a POI mouse model, stimulation of the vagus nerve via the close contact between MMs and enteric cholinergic neurons reduced intestinal inflammation [70]. Signals from gut microbiota could affect the crosstalk between MMs and the ENS, thus altering gastrointestinal motility [71]. MMs send signals to the ENS by secreting BMP2. The ENS promotes the maintenance of macrophages through macrophage growth factor colony-stimulating factor 1. MMs and intrinsic enteric neurons communicate bidirectionally and support each other’s functions [54]. Neurons can label specific phenotypes to macrophages in the niche, and neuronal signals can signal macrophages to preserve neuronal networks in response to inflammation or infection. The mechanism by which BMP2 affects the function of steady-state or differentiated Enteric neurons remains unclear; however, the role of BMP receptor signaling in regulating microtubule stability, rapid axonal transport, synaptic growth, and stability of axons has been established [71]. Antibiotic treatment reduces the expression of BMP2, the number of MMs in neurons, and signal transduction through the BMP receptor and colony-stimulating factor 1 expression. These changes lead to alterations in gastrointestinal motility [72].

Macrophages have M1 and M2 phenotypes, which produce inflammatory and anti-inflammatory cytokines, respectively [73]. Abdominal surgery activates M1 macrophages increaseing the expression of proinflammatory cytokines [74]. Shifting from the anti-inflammatory M2 to the proinflammatory M1 inactivates enteric neurons and enteric neural stem cells and delays intestinal transit [75]. In antibiotic-treated mice, the expression of M1 macrophage markers increased, whereas that of anti-inflammatory M2 markers decreased. An increase in M1 macrophages is related to prolonged gastrointestinal transport time [76]. In sterile mice transplanted with symbiotic bacteria, M2 macrophages migrating into the muscularis of the gastrointestinal tract may accelerate gastrointestinal motility. Therefore, a low M2 macrophage count was associated with inhibited intestinal motility in mice. However, the underlying mechanisms remain unclear. Intestinal transplantation or major abdominal surgery can lead to the translocation of gut microbiota and the release of pro-inflammatory cytokines from M1-like macrophages. This could result in circulating leukocyte recruitment, thus reducing smooth muscle function and impairing intestinal peristalsis by releasing nitric oxide [76].

T lymphocytes interacts with gut microbiota to regulate intestinal homeostasis, plays an important role in POI. CD4 knockout alleviates the damage to gut motility caused by abdominal surgery in mice [77]. In a study, *Escherichia coli* affect the differentiation of Th17 and Treg cells to destroy the balance of Th17/Treg cells, then induce intestinal inflammation, and damage gut motility [78]. Amphiregulin derived fromTh17 cell promotes intestinal fibrosis by activating mTOR and MEK in intestinal myofibroblasts,which may lead to POI [79]. In addition, CD8 T cells plays an important pathogenic role in gut motility disorders by disrupting intestinal neurons [80].

In a POI mouse model, gastrointestinal surgical injury resulted in the local production of the pro-inflammatory mediators IL-12 by dendritic cells and interferon-γ by memory T helper type 1 cells, which activated macrophages to express iNOS. The nitric oxide produced by iNOS paralyzed intestinal muscle cells, leading to POI [64, 77]. In addition, a recent study showed that a deficiency in CD103 + CD11b + dendritic cells in the intestinal myometrium of mice reduced the pathogen-inducible iNOS in monocytes and macrophages, thereby improving POI [81].

In conclusion, gut microbiota imbalance, increases the intestinal inflammatory response, and neuron–macrophage interaction disorder may affect the homeostasis of the ENS, thereby leading to gastrointestinal dysfunction.-

### Gut microbiota affect the ENS by regulating 5-HT

The 5-HT neurotransmitter plays a vital role in the functions of the ENS and gastrointestinal system, further regulating intestinal secretion and motility [82]. As an important paracrine signal molecule, 5-HT affects intestinal epithelial cell secretion and the intestinal barrier function through the G protein-coupled receptors on adjacent cells [83], directly and indirectly regulating intestinal motility. As an intestinal neurotransmitter regulating the neurokinin receptor, substance P (SP) controls the intestinal motor and sensory functions and strongly promotes smooth muscle contraction [84]. Under mechanical or chemical stimulation, enterochromaffin cells (ECs) release 5-HT, which upregulates the release of SP in afferent nerve fibers by acting on the 5-HT3 receptor [83], and activates the neurokinin-1 receptor, increasing the SP-mediated motor response. Approximately 10% of 5-HT is produced in the ENS and central nervous system, which stimulates the local intestinal nerve reflex, triggers secretion and propulsion and acts on the motor vagal afferent nerve to regulate contractile activity [85]. Using a POI model of guinea pigs, one study has shown that treatment with 5-HT4 agonists before surgery can significantly accelerate intestinal movement and improve POI [86]. At the same time, a meta-analysis found that treatment with 5-HT agonists can promote intestinal recovery post-surgery [87]. Mechanistically, activated 5-HT4 promotes intestinal movement by activating enteric cholinergic neurons and inhibiting the inflammatory reaction of the intestinal muscle layer [88, 89].

Gut microbiota can regulate gastrointestinal peristalsis by affecting the synthesis of 5-HT [90, 91]. In mice, spore-forming bacteria, such as *Clostridium*, promote the synthesis of 5-HT in colonic chromaffin cells and affect intramuscular neurons and gastrointestinal motility by upregulating 5-HT receptors on submucosal neurons and increasing 5-HT levels in the blood [85]. In germ-free mice, the levels of 5-HT in the colon and feces and the expression of tryptophan hydroxylase 1 (TPH1) in the colon were reduced, and gut microbiota promoted 5-HT biosynthesis by increasing the expression of TPH1 in ECs [92]. Concurrently, data on clearance of the gut microbiota of specific pathogen-free mice using antibiotics showed that intestinal microorganisms had a sustained effect on 5-HT synthesis by regulating the function of ECs [93]. In specific probiotics (EcN-5-HT) colonization experiments, colonization shortened gastrointestinal transit time, increased fecal excretion, and improved gastrointestinal motility by increasing the level of 5-HT [91]. In brief, various studies have shown that gut microbiota affects gastrointestinal motility by regulating 5-HT levels [91, 94].

The mechanism by which gut microbiota affect postoperative intestinal motility by regulating 5-HT has attracted increasing research attention. Recent studies have shown that gut microbiota affect gastrointestinal motility by increasing the biosynthesis of 5-HT in ECs through fermentation end-products, such as SCFAs and Bas [94].

### Gut microbiota metabolites affect intestinal motility by regulating the ENS

When the barrier function of the gut is impaired, the ENS may be exposed to the metabolites produced by gut microbiota affecting intestinal motility. The metabolites of gut microbiota can promote the synthesis of various neurotransmitters and regulate the secretion of signal molecules. Intestinal remodeling after abdominal surgery alters the metabolism of gut microbiota [65].

BAs exert bacteriostatic effects, and microorganisms transform primary BAs to produce secondary BAs. Liver injury caused by surgery reduces the level of BAs [95]. BAs can prevent intestinal bacterial overgrowth, maintain barrier function, promote gastrointestinal peristalsis by activating the G protein-coupled receptor (TGR5), and affect intestinal movement by affecting the mechanism of Ret signal transduction in the ENS [95]. However, BAs release nitric oxide and inhibit movement by activating TGR5 in inhibitory motor neurons. Increased BA levels upregulate the expression of NOS and TGR5 in the gastroenteric nerve plexus delaying gastric emptying [96, 97]. This finding contradicts the conclusion that BAs promote intestinal peristalsis; thus, this issue requires further research.

SCFAs can inhibit the growth of pathogens, and their metabolic activity is related to various gastrointestinal functions, such as intestinal motility and mucus secretion, through nerve and muscle stimulation [98].

A clinical study reported that probiotics could promote the production of SCFAs, especially by increasing acetate, butyrate, and propionate, thereby reducing postoperative intestinal complications and the occurrence of POI [99]. A recent study showed that butyrate plays a regulatory role in microbiota TLR-dependent sensing [100]. Microorganisms are involved in intestinal motility by affecting the release of peptide YY andglucagon-like peptide-1 from enteroendocrine L-cells via stimulating TLRs [101]. These secretions could enhance the propulsion of the colon, increase the contraction of colonic circular muscles, and improve gastrointestinal motility by promoting the development of cholinergic and nitrate neurons [102, 103]. Butyric acid induces changes in the neural plasticity of the ENS, leading to neuro proliferative changes in intestinal myenteric and submucosal neurons and enhancement of colonic motility [102]. Dysbiosis after surgery led to a significant decrease in butyric acid and SCFAs and an increase in the venous pressure in the intestine [104]. All these changes may decrease intestinal motility, impair the removal of harmful bacteria, and reduce theanti-inflammatory response. Moreover, changes in gut microbiota after surgery might lead to insufficient decomposition of dietary components, such as lipids and complex polysaccharides [105], leading to a reduction in SCFA levels and an imbalance in the ENS system, resulting in intestinal dyskinesia.

## Gut microbiota and their metabolites affect postoperative intestinal motility by regulating the aryl hydrocarbon receptor (AHR)

A recent study showed that the AHR signal in the intestinal nerve circuit connects gut microbiota and intestinal nerve function and plays an important role in regulating the intestinal motor function [106]. Specific deletion of AHR neurons or overexpression of its negative feedback regulator Cytochrome P450 Family 1 Subfamily A Member 1 can inhibit colonic peristalsis. In contrast, gut microbiota and their metabolites can combine with AHR to activate the immune system, enhance the intestinal epithelial barrier, and stimulate gastrointestinal peristalsis [107]. In a control experiment involving specific pathogen-free mice and germ-free mice [108], the expression of AHR in the colon tissues of germ-free mice and antibiotic-treated mice decreased, while the frequency of colonic transitional motor complexes decreased, and intestinal peristalsis slowed. Depletion of microorganisms reduces the number of available AHR ligands, decreases the excitability of enteric neurons, and significantly prolongs intestinal transport time. These findings suggest that gut microbiota can induce AHR expression in colon tissues, thereby regulating movement of the intestinal nerve circuit.

In addition, metabolites produced due to tryptophan decomposition by gut microbiota are important signal molecules among microbiota and in host–microbiota crosstalk and may maintain homeostasis in the gastrointestinal system. Tryptophan metabolites can enhance the intestinal epithelial barrier function, reduce inflammation, regulate glucagon-like peptide-1 secretion, and affect gastrointestinal peristalsis. The metabolites resulting from the bacterial decomposition of tryptophan were identified as AHR ligands, which may activate AHR and affect cytokines [107]. Moreover, indoleacetic acid and tryptamine, produced in the metabolism of tryptophan, attenuated the response of proinflammatory cytokines in mouse macrophage cultures in an AHR-dependent manner [109]. The effects of tryptophan metabolites on cytokines depend on the activation of AHR, and AHR signal transduction can modify the TLR-regulated response in dendritic cells [110]. However, SCFAs might also enhance the gene induction of AHR, in which acetate, propionate, and butyrate improve the response induced by the AHR ligand. Gut microbiota and their metabolites can activate the AHR pathway in a ligand-dependent manner, subsequently regulating the differentiation of AHR to promote or control the release of anti-inflammatory factors. However, preoperative intestinal cleaning and intraoperative gastrointestinal reconstruction of patients undergoing gastrointestinal surgery may result in changes in gut microbiota; however, whether gut microbiota could participate in postoperative intestinal motility recovery through the AHR pathway has not been determined and should be further investigated.

## Probiotic supplements to improve postoperative intestinal motility injury

Surgery may increase the number of pathogenic bacteria in the intestine and decrease the proportion of beneficial bacteria, such as Lactobacillus and Bifidobacterium; administration of antibiotics before surgery reduces the abundance of gut microbiota, leading to dysbiosis [111, 112]. Some probiotic strains may affect intestinal motility and secretion by altering the intraluminal environment; therefore, these strains might be beneficial to patients with postoperative intestinal motility injuries. For instance, a study has shown that specific probiotics may help decrease the gut transit time and improve constipation-related symptoms in patients [113]. The microbiota–gut–brain interaction and regulation of probiotics are considered new therapeutic tools for the treatment of POI [114]. Pretreatment with probiotics before surgery increases the abundance of beneficial bacteria, promotes butyrate production, and stimulates excretion [23]. A meta-analysis study analyzed the time of the initial postoperative flatulence, the initial defecation, days of the first solid diet, incidence of abdominal distension, and incidence of postoperative intestinal obstruction and found that probiotics supplements reduced the incidence of abdominal distension (RR, 0.62) and POI (RR, 0.47) [115]. In another randomized, double-blind, placebo-controlled clinical study of 100 adults with slow transit constipation [116], supplementation of synbiotics increased stool frequency, improved stool consistency, reduced intestinal transit time, improved intestinal motility, and relieved constipation. In a prospective, randomized controlled trial targeting patients undergoing craniotomy, oral supplementation with probiotics shortes the time of first stool and flatus [117]. *Lactobacillus rhamnosus* GG promotes passing gas and the first postoperative stock of patients suffering pylorus preserving pancreaticoduodenectomy [118]. These results indicate that probiotics can improve intestinal motility, and the nervous system may mediate the beneficial effects. Postoperative application of some probiotics may affect intestinal motility and secretion; however, the complex interactions of the different probiotics and strains with the ENS and intestinal motility, along with the specific mechanisms of action, need to be further studied.

In conclusion, preoperative administration of antibiotics, opioid anesthetics, and injury as a result of gastrointestinal surgery leads to disorders in gut microbiota and their metabolites, which can affect the neuromuscular regulation of gastrointestinal motility through the release of inflammatory cytokines or neurotransmitters or direct activation of signaling pathways in intestinal myometric neurons. Reducing intestinal tissue damage during surgery, shortening anesthesia time, avoiding excessive mechanical bowel preparation, and subsequently reducing gastrointestinal microbiota disorders are effective ways to improve the management of POI. The clinical application of some probiotics may provide a way to treat postoperative intestinal motility. In addition, based on neuroanatomy, neuroprotective mesenteric and intestinal tissue cutting may help improve and alleviate POI.

## Data Availability

Not applicable.

## Abbreviations

POI: Postoperative ileus
BAs: Bile acids
SCFAs: Short-chain fatty acids
SP: Substance P
5-HT: 5-Hydroxytryptamine
IL: Interleukin
ME: Muscularis externa
ENS: Enteric nervous system
TLRs: Toll-like receptors
LPS: Lipopolysaccharides
iNOS: Inducible nitric oxide synthase
MCs: Mast cells
MMs: Muscle macrophages
BMP2: Bone morphogenetic protein 2
ECs: Enterochromaffin cells
TPH1: Tryptophan hydroxylase 1
AHR: Aryl hydrocarbon receptor
